# Effect of Curing on Micro-Physical Performance of Polypropylene Fiber Reinforced and Silica Fume stabilized Expansive Soil Under Freezing Thawing Cycles

**DOI:** 10.1038/s41598-020-64658-1

**Published:** 2020-05-06

**Authors:** Nitin Tiwari, Neelima Satyam, Kundan Singh

**Affiliations:** Discipline of Civil Engineering, Indian Institute of Technology Indore, Indore, India

**Keywords:** Engineering, Materials science

## Abstract

This study presents the micro-physical investigation of polypropylene (PP) fiber-reinforced, and silica fume (SF) stabilized expansive soil (BC) subgrade. The coupling effect of soil, PP fiber, and SF has been evaluated under the freezing-thawing (F-T) cycle to assess the durability of treated BC Soil. The curing method and duration staggeringly influence the strength of SF treated BC soil; therefore, three different curing method, i.e., moisture-controlled curing (MC), gunny bag curing (GC), and water submerged curing (SC) to a period of 7, 14, and 28 days were considered. The BC soil has been reinforced with 0.25%, 0.50%, and 1.00% PP fiber and stabilized with 2%, 4%, 6% and 8% SF. The physical, chemical, and microstructural properties were determined before and after 2,4,6,8,10 F-T cycles. With the increase in SF content, the unconfined compressive strength of the expansive soil has been increased due to the formation of Calcium Silicate Hydrate (C-S-H) gel. The chemically inert, hydrophobic, non-corrosive nature, and higher tensile strength of PP fiber, it has a higher potential to reinforce the BC soil for durability under tensile failure. This research confirms the possibility of incorporating SF and PP Fiber in road work applications, with significant environmental benefits.

## Introduction

The infrastructure development like paved structure, building, dams involves exhaustive use of natural resources and has a serious concern to the environment^1^. Industrialization is proliferating in developing countries such as India; hence, the disposal of industrial waste became a severe threat^2^. A paved structure (highway, airfields, lawns) is the composite structure that usually consists of a surface layer, base layer, and subgrade layer. The durability of paved structures majorly affected by the performance of the subgrade material. The soft soil is found in the semi-arid and arid regions of the world. The soft soil is also termed as expansive soil or black cotton soil. About 3.50 million square kilometers of vertisols, clayey soils rich in smectite, are spread worldwide over 60 countries and regions^3,4^. Expansive soil is found in Australia (0.80 million square kilometers), India (0.73 million square kilometers), China (0.60 million square kilometers), Sudan (0.50 million square kilometers), southern and western states of United States (0.13 million square kilometers), Chad, Cuba, Egypt, Ethiopia, Ghana, Puerto, Rico, and Taiwan^5^.

The layered structure of the clay minerals and cations adsorbed for the charged equilibrium induced swelling-shrinkage in expansive soil^6^. A higher nonrecurring swelling-shrinkage behavior of expansive soil culminate damages to the built infrastructure^7^. The damages caused by the expansive soil can be observed around the world; approximately $7–$ 9 billion per year economic losses have been reported alone in the United States^8^. The major deterioration was espied on the canals, pathways, buried pipelines, and paved structures. The numerous remedies were successfully demonstrated to improve the engineering behavior of the expansive soil, such as chemical alternation additive^9–13^, moisture control^14,15^, application of adequate surcharge pressure^16^, Biocementation^17^, biochemical^18–20^, and Geosynthetic^21–23^.

The chemical stabilization is a substantiate quick-fix method to improve the shear strength and also reduces the swelling shrinkage nature of expansive soil^24–27^. Nonetheless, there are substantial problems for the long-term viability of these approaches, depending on soil clay mineralogy, ecological variations in clay^19^. The chemical stabilization method exponentially increases the compressive strength of the expansive soil; however, it dispenses the minimal effect on tensile strength. The several chemical stabilization agents can be grouped as conventional (lime, cement)^28^, industrial waste by-products (fly ash, quarry dust, phosphorus gypsum, slag)^29–33^, and non-traditional (sulfonated oils, potassium compounds, polymers, enzymes, ammonium chlorides)^30,34^. Cement has used as a conventional chemical stabilizer to improve the engineering properties of soil, such as strength and durability^35^; however, it causes global warming due to CO_2_ emission^36,37^. Therefore, to reduce the carbon emission caused due to cement required its substitute^38^. The various industrial waste by-products have manifested to chemically stabilized the expansive soil. A silica fume is one of the non-expansive industrial waste materials that significantly improves the strength and reduces upward swell pressure in expansive soil subgrades^26^. Nevertheless, SF is a byproduct of industrial waste material, and its open disposal is a serious concern for air pollution. The open disposal can pose various health problems to the locacality^39^. The silica fume majorly consists of amorphous silica (SiO_2_). The higher specific surface area, cementation effect, and pozzolanic activity of SF attributed higher potential to use as an alternative of cement and lime^40–44^.

The expansive soil stabilized by cementation material has suffered from brittle shear failure. The tensile strength of the expansive soil is an essential mechanical property in the structural design. Various attempts have been made to enhance the mechanical properties of chemically stabilized soil. The inclusion of natural and/or synthetic fiber has shown substantial improvement against tensile failure^45^. The various fibers, i.e., polypropylene fiber^46–49^ basalt fiber^50,51^, coir fiber^52^, sisal fiber^53^, carpet waste fiber^54^, glass fiber^55^, waste rubber fiber^56^, polyvinyl alcohol fiber^57^, kenaf fiber^58^ palm fiber^59^, and polyester fiber^60^ helps in improving the tensile strength of the chemically stabilized expansive soil. Polypropylene fiber is highly acid and salt resistant, and its higher tensile strength shows a higher potential to be used as microfine reinforcement^29^. The chemically inert, hydrophobic, and non-corrosive nature of PP fiber also indicates its effective utilization in the soil reinforcement^61,62^.

The various advantages of the PP fiber with silica fume stabilized expansive soil has been presented in the literature. Nitin *et al*. 2019, explained the effect of SF and PP fiber inclusion with BC soil to reduce the upward swell pressure and its mitigation mechanism^63^. However, the coupling behavior of soil, SF, and PP fiber at the microstructural level and for durability assessment is not present. Yixian *et al*. 2019, presented improvement in the mechanical behavior of wheat straw fiber reinforcement and lime stabilization clayey soil at the microstructural level^64^. The objective of this work is to investigate the micro-physical performance of SF, and PP fiber treated expansive soil under the freeze-thaw cycle. The microstructural analysis of treated and untreated samples have been carried out by Scanning Electron Microscopy (SEM) and Energy-dispersive X-ray (EDX) spectroscopy. The inclusion of SF forms hydration, which alters the chemical bonds and mineralogy of the BC soil. The Attenuated Total Reflection Fourier-Transform Infrared (ATR-FTIR) spectroscopy and X-ray Diffraction (XRD) has been performed to assess the alterations. The treated BC soil specimens were tested for mechanical strength by conducting the Unconfined Compression Strength (UCS), Split Tensile Strength (STS) Ultrasonic Pulse Velocity (UPV). The chemical changes were evaluated using calcite content (CCt), pH, and electrical conductivity (EC).

## Materials and methods

### Material

The soil used in this study was collected from the Malwa plateau site inside the Indian Institute of Technology Indore, Madhya Pradesh, located in the central part of India. The samples have been excavated at a depth of 1.5 m to 2.0 m from ground level. The major constitutes of soil clay (71.5%), silt (24.5%), and sand (4.0%) were observed as per grain size distribution. The Atterberg’s limits of the expansive soil were also determined (liquid limit at 89%, plastic limit at 47%, and shrinkage limit at 11%). As per the Unified Soil Classification System (USCS), the soil has been classified as inorganic clay with high plasticity (CH). The maximum dry density (MDD) and optimum moisture content (OMC) have been found to be 17.65 kN/m^3^ and 19.20%, respectively. A 120% Free Swell Index (FSI) has been observed for 7 days; this indicates the higher potential of swelling in soil. Table 1 summarizes the results of the index properties of expansive soil considered in the present study. The index properties of the expansive soil have been evaluated as per Bureau of Indian Standard IS 2720.

**Table 1:** Index properties of Expansive soil.

Property	Value	Test Standard
MDD (kN/m^3^)	17.65	IS 2720:1980 (Part VII)^95^
OMC (%)	19.2	IS 2720:1980 (Part VII)^95^
Liquid limit (%)	89	IS 2720:1985 (Part V)^95^
Plastic limit (%)	47	IS 2720:1985 (Part V)^95^
Plasticity index (%)	42	IS 2720:1985 (Part V)^95^
Shrinkage limit (%)	11	IS 2720:1972 (Part VI)^95^
Clay (%) Silt (%) Sand (%)	71.5 24.5 4.0	IS 2720:1985 (Part IV)^95^
Specific gravity	2.78	IS 2720:1980 (Part III)^95^
Free swell index (%)	120	IS 2720:1977 (Part XL)^95^
USCS soil classification	CH	IS 2720:1985 (Part IV)^95^

PP fiber is produced from a polymeric material and waste plastics. The PP fiber is highly acid, and salt resistance. Its higher tensile strength shows a higher potential to use the material as microfine reinforcement. The chemically inert, hydrophobic, and non-corrosive nature of PP fiber also indicates its effective utilization in the soil reinforcement due to low susceptibility of absorption or reaction with soil moisture or leachate. The polypropylene (PP) fibers of an average length of 12 mm have been considered. The technical specification of the considered PP fiber is tabulated in Table 2.

**Table 2:** Properties of polypropylene fiber considered.

Property	Value	Test Standard
Strength (kN/mm^2^)	0.67	ASTM D747^96^
Specific gravity	0.91	ASTM D792^97^
Modulus of Elasticity (kN/mm^2^)	4.0	ASTM D638^98^
Melting temperature (°C)	165	ASTM D7138^99^
Ignition temperature (°C)	600	ASTM E3020^100^
Bulk density (kg/m^3^)	910	ASTM D3800^101^
Loosen density (kg/m^3^)	250–430	ASTM D3800^101^
Length of fiber (mm)	12 mm	ASTM D5103-07^102^
Aspect Ratio	4	ASTM D5103-07^102^
Salt and acid effect	Excellent	ASTM C1012 -04^103^ ASTM C563-07^104^

The cementation material used in this study is silica fume (SF) and its chemical compositions and mechanical properties are tabulated in Table 3. It is an industrial waste derived from silicon metal or ferrosilicon alloy. The SF majorly consists of amorphous silica (SiO_2_). The higher specific surface area, cementing effect, and silica fume pozzolanic activity exhibit a higher potential to use as a cement and lime alternative.

**Table 3:** Silica fume properties considered.

Property	Value	Test Standard
Density, (Mg/m^3^)	92.25	ASTM C1240-20^105^
Specific Gravity	2.24	ASTM C1240-20^105^
SiO_2_ (%)	98.87	ASTM E1621^106^
Al_2_O_3_(%)	0.02	ASTM E1621^106^
Fe_2_O_3_ (%)	0.01	ASTM E1621^106^
K_2_O (%)	0.08	ASTM E1621^106^
CaO (%)	0.23	ASTM E1621^106^

### Sample preparation

The four types of samples have been prepared to investigate the effect of curing methods on freezing-thawing cycle of PP fiber-reinforced, and silica fume stabilized expansive soil: (1) an untreated sample; (2) an SF stabilized sample; (3) a PP Fiber-reinforced samples, and (4) a PP fiber-reinforced and SF stabilized sample. The expansive soil has been reinforced with 0.25%, 0.50%, and 1.00% PP fiber and stabilized with 2%, 4%, 6%, and 8% SF as reported by Tiwari and Satyam (2019)^63^. The expansive soil samples were air-dried until a stable state was reached. Several mixtures were prepared by mixing the various percentages of water to achieve a uniform mixture of all ingredients.

The behavior of BC soil is affected by optimum moisture content (OMC) and maximum dry density (MDD)^65^. The specimen was therefore prepared at the MDD and OMC. The 4.75 mm passed dry mixture of 105–110 °C oven drived BC soil, SF (2.0%, 4.0%, 8.0%), and PP fiber (0.25%, 0.50%, 1.00%) was prepared. The dry mix was maintained at 27 ± 2 °C and 65 ± 5% humidity in the environmental chamber to preserve the soil mass constant temperature and uniform moisture content.

**Figure 1:**
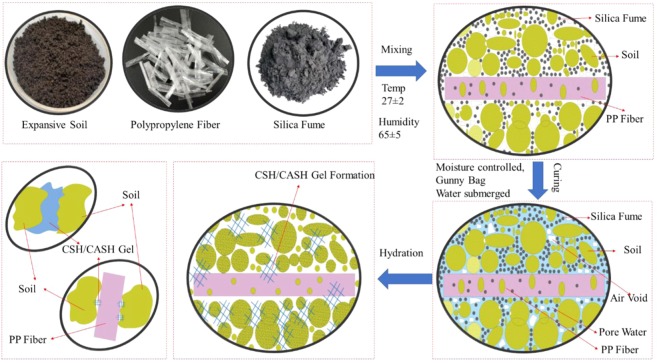
Schematic plan of the sample preparation method of soil, SF, and PP fiber.

The 38 ± 2 mm diameter 76 ± 4 mm length specimens of the various mix have been papered with the statically simple compaction method. The compaction of the sample was carried out using an automatic soil compactor to avoid the change in energy level of a hammer. The statically compacted specimens have been kept for three different curing methods, i.e., MC, GC, SC; each to a period of 7 14, and 28 days. The maximum duration for the curing has been finalized since the various studies reported that the basic hydration reaction reached stable in 28 days curing period^66^. Figure 1 shows the schematic plan of the sample preparation method of soil, SF, and PP fiber.

The treated specimens were grouped into three categories to investigate the effect of different types of curing methods. The prepared specimens have been covered with the latex membrane for the curing process. The specimens have been kept in the environmental chamber at the 20 ± 2 °C and 100 ± 5% humidity for the MC curing. The specimens have been warped in the wet gunny bag for the GC and for SC the specimens have been submerged in the water tank at room temperature.

### Chemical and microstructural analysis

The microstructural components of the PP fiber-reinforced SF stabilized expansive soil was studied using SEM, ATR-FTIR, XRD, and EDX analysis. The selected specimens after the UCS and STS test were taken for the SEM and EDX analysis. The freeze-cut-dying method was used to preparing the soil specimen for the microstructural analysis. This method can preserve the original soil microstructure and minimize flaws such as erroneous orientation and false void, which occur in the SEM image^67^. The soil specimens have been crushed and coated with (Cu) copper material to reduce the charging effect on the samples during SEM analysis. To remove the water and organic contamination, soil specimen has been degreased and dried in vacuum. The required amount of the sample has been pasted on the carbon tape and then surface coated by Cu material^68^.

Since the PP fiber is used as the reinforcement material and does not have any influence on the chemical structure of the stabilized soil, hence the ATR-FTIR and XRD analysis have been carried out only on SF stabilized specimens with different curing method. The KBr pellet technique has been adopted for the FTIR. IR Spectral characterization of the pellet was performed from 400 cm^−1^ to 4000 cm^−1^ using ATR-FTIR spectrometer (PerkinElmer) equipped with a potassium bromide beam splitter. The XRD test has been performed on the peat sample of size less than 75 microns. The SF content highly influences the chemical alternations in the expansive soil. Electrical conductivity, pH, and Calcite content test have been conducted to determine the chemical alternation in expansive soil due to addition of SF.

### Mechanical property analysis

A total of 408 specimens were prepared using the aforementioned method. The detailed mix design plan with the test section, and several specimens tested are tabulated in Table 4. To understand the durability characteristics of the untreated and treated BC soil, the specimen was kept in the freezing-thawing chamber. After completion of each curing cycle i.e., 7, 14, 28 days of PP reinforced SF stabilized expansive soil specimens. The freezing-thawing test has been conducted in the closed system. Before F-T analysis, the treated and untreated specimens were warped with transparent plastic to reduces the atmospheric interaction. The F-T cycle has been set at freezing temperature −20 °C for the first 12 hrs and then thawing temperature +20 °C to another 12 hrs^69^. The F-T test has been carried out up to 10 cycles, considering the expansive soil, which shows stable deformation and strength characteristics after an adequate F-T cycle^70^. After every two F-T cycles i.e., 0, 2, 4, 6, 8, 10, the one specimen has been taken out from each sample type and tested under UPV, UCS, STS experiments

**Table 4:** Experimental Test Program.

Sample ID	Material Type (% by weight)			Test Performed*	Number of Specimen Tested^#^ = 408
	BC	SF	PP		
BC	100.0	0.0	0	UCS, STS, UPV, CCt,pH,EC	24
BC + 2%SF	98.00	2.0	0	UCS, STS, UPV, CCt,pH,EC	24
BC + 2%SF + 0.25%PP	97.75	2.0	0.25	UCS, STS, UPV	24
BC + 2%SF + 0.50%PP	97.50	2.0	0.50	UCS, STS, UPV	24
BC + 2%SF + 1.00%PP	97.00	2.0	1.00	UCS, STS, UPV	24
BC + 4%SF	96.00	4.0	0	UCS, STS, UPV, CCt,pH,EC	24
BC + 4%SF + 0.25%PP	95.75	4.0	0.25	UCS, STS, UPV	24
BC + 4%SF + 0.50%PP	95.50	4.0	0.50	UCS, STS, UPV	24
BC + 4%SF + 1.00%PP	95.00	4.0	1.00	UCS, STS, UPV	24
BC + 6%SF	94.00	6.0	0	UCS, STS, UPV, CCt,pH,EC	24
BC + 6%SF + 0.25%PP	93.75	6.0	0.25	UCS, STS, UPV	24
BC + 6%SF + 0.50%PP	93.50	6.0	0.50	UCS, STS, UPV	24
BC + 6%SF + 1.00%PP	93.00	6.0	1.00	UCS, STS, UPV	24
BC + 8%SF	92.00	8.0	0	UCS, STS, UPV, CCt,pH,EC	24
BC + 8%SF + 0.25%PP	91.75	8.0	0.25	UCS, STS, UPV	24
BC + 8%SF + 0.50%PP	91.50	8.0	0.50	UCS, STS, UPV	24
BC + 8%SF + 1.00%PP	91.00	8.0	1.00	UCS, STS, UPV	24

^*^All the specimens have been kept for three different curing method i.e., moisture controlled, gunny bag, and water submerged to a period of 7, 14 and 28 days.

^#^To understand the repeatability of test result, all the experiments were carried out thrice on treated and untreated BC soil.

**Figure 2:**
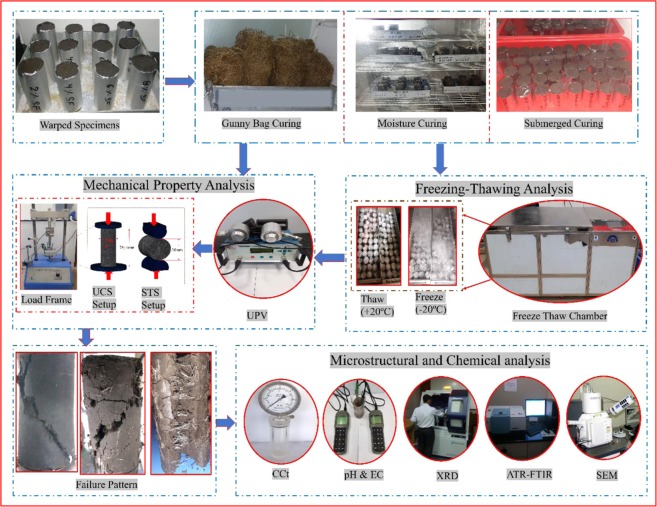
Detailed Experimental procedure to assess the Micro-Physical Coupling effect of PP fiber Reinforced and SF stabilized Expansive soil.

The reproducing of the experimental results is highly desirable; to understand the repeatability of test results all the experiments were carried out thrice on treated and untreated BC soil. The findings of repeatability indicate 9.8% as the highest standard deviation. All experimental results are presented on average for each experiment. The detailed experimental methodologies for stabilizing the BC soil shown in Fig. 2.

## Results and Discussion

The effect of different curing method on PP fiber-reinforced and SF stabilized expansive under the freezing-thawing cycle have been evaluated. The experimental program has been conducted on 408 treated and untreated expansive soil specimens. The test results with varying curing methods and curing period have been compiled in the following section with detailed discussion.

### Effect of the curing method on UCS value of treated and untreated BC soil

**Figure 3:**
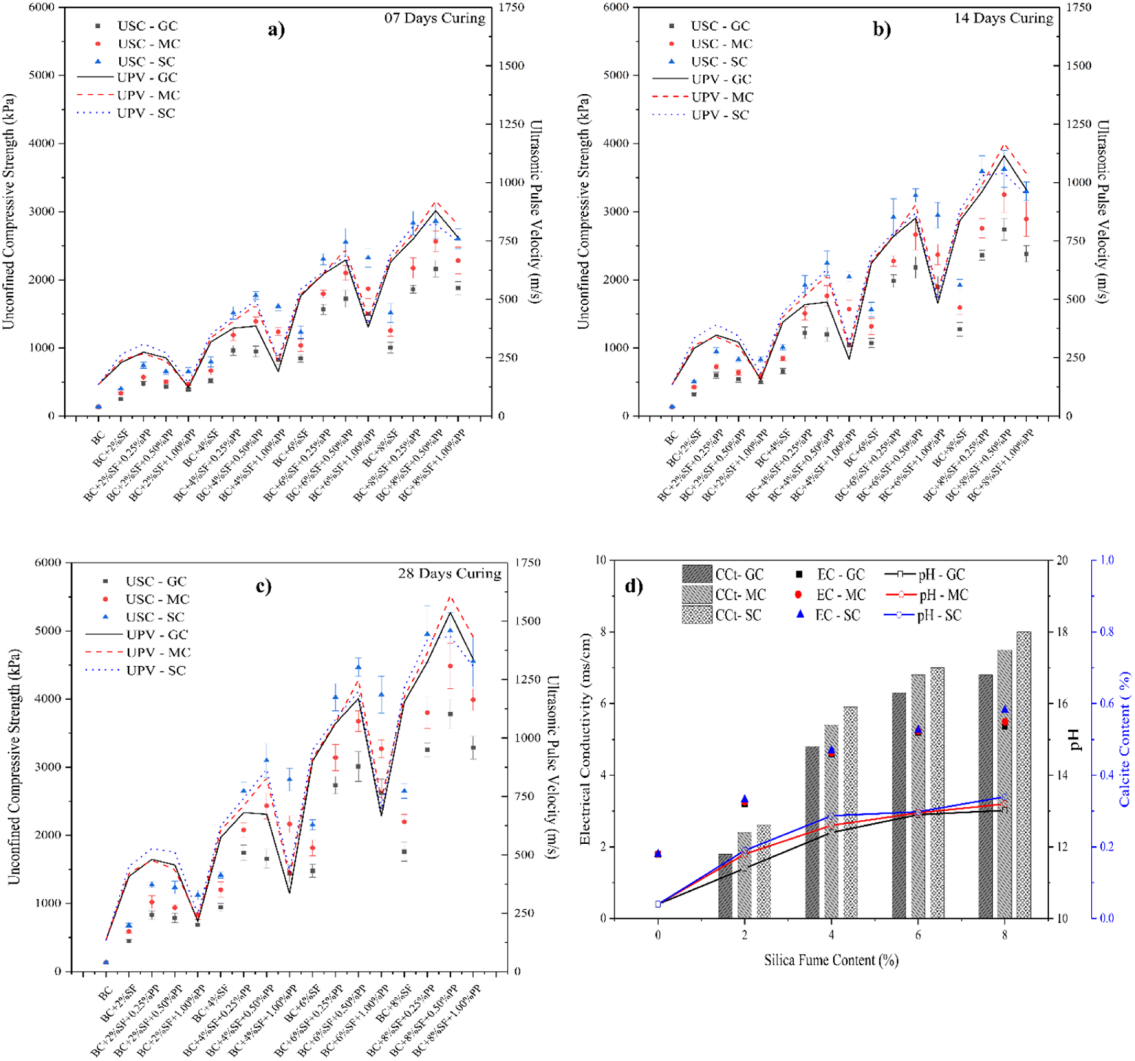
Effect of curing method on (**a**) 07 days curing (**b**) 14 days curing (**c**) 28 Days curing (**d**) chemical analysis.

The effect of the curing method on the unconfined compressive strength of the PP fiber-reinforced and SF stabilized expansive soil is shown in Fig. 3. The results of the GC, MC, and SC with 7, 14, and 28 days has been presented. The improvement in the unconfined compressive strength can be significantly observed. A similar failure pattern has been observed in all three curing period. However, the strength values have been increased exponentially. It has been observed that with the increment in the SF content, the compressive strength increased. However, with the addition to the higher concentration of the PP fiber content, the compressive strength has been reduced. The increase in the compressive strength with the inclusion of SF can be attributed to the formation of Calcium silicate hydrate (C-S-H). The electrical conductivity, calcite content, and pH value test have been conducted to understand the chemical reaction of the SF with expansive soil, and test results were presented in Fig. 3(d). The value of EC is increased continuously by increasing the percentages of SF, which confirms that the chemical reaction takes place with the inclusion of SF with BC soil and water. It is well established that the rise in atmospheric temperature will improve the hydration of cement^71,72^. The EC values increase significantly with the addition of SF content. The pH values have also been increased with increasing the SF content, which signifies the changes in the nature of BC soil-SF suspension and confirms the chemical alteration in the treated specimen.

The increment in the pH value increases the shear strength parameter of the soil specimen^73^. The higher specific surface area of the soil matrix makes it an effective filler. This reduced the volume of soil voids and induced the homogeneous production of CSH / CASH gel, i.e., sound microstructure^74,75^. The significant improvement in the CCt is also observed with the addition of the SF. Increment in the CCt can be attributed to the formation of the C-S-H gel^20^. Among all curing methods, SC curing shows the highest improvement in UCS values; however, the effect of the GC curing method is lowest. It can be ascertained that both the method i.e., SC and MC show promising improvement. Calcium silicate hydrate (C-S-H) gel is produced when SF content associated with the water while undergoing the pozzolanic reaction, as shown in Eq. 1. The chemical reaction of SF and calcium makes more brittle and steeper treated specimens. The brittle behaviors of SF stabilized soil exponentially increase the UCS value.

1 $$C{a}^{2+}+O{H}^{-}+soluble\,silica\to calcium\,silicate\,hydrate$$

The maximum UCS has been obtained as 2862.13 kPa for a sample ID BC + 8%SF + 0.50%PP at 7 days curing as shown in Fig. 3(a). A similar trend has been observed for 14 days and 28 days, as shown in Fig. 3(b,c). The maximum UCS has been observed as 3627.54 kPa, and 5003.51 kPa for the 14 days and 28 days, respectively, for the sample ID BC + 8%SF + 0.50%PP. However, it can also be observed that at 7 days curing period, the maximum UCS for GC, MC, SC are obtained as 2161.20 kPa, 2565.43kPa, and 2862.13 kPa, respectively. This result deduces the effect of curing on the same sample. From the result, it can be ascertained that the effect of the submerged curing is higher than the moisture-curing and gunny bag curing.

### Effect of curing method on chemical alteration of treated and untreated BC soil

**Figure 4:**
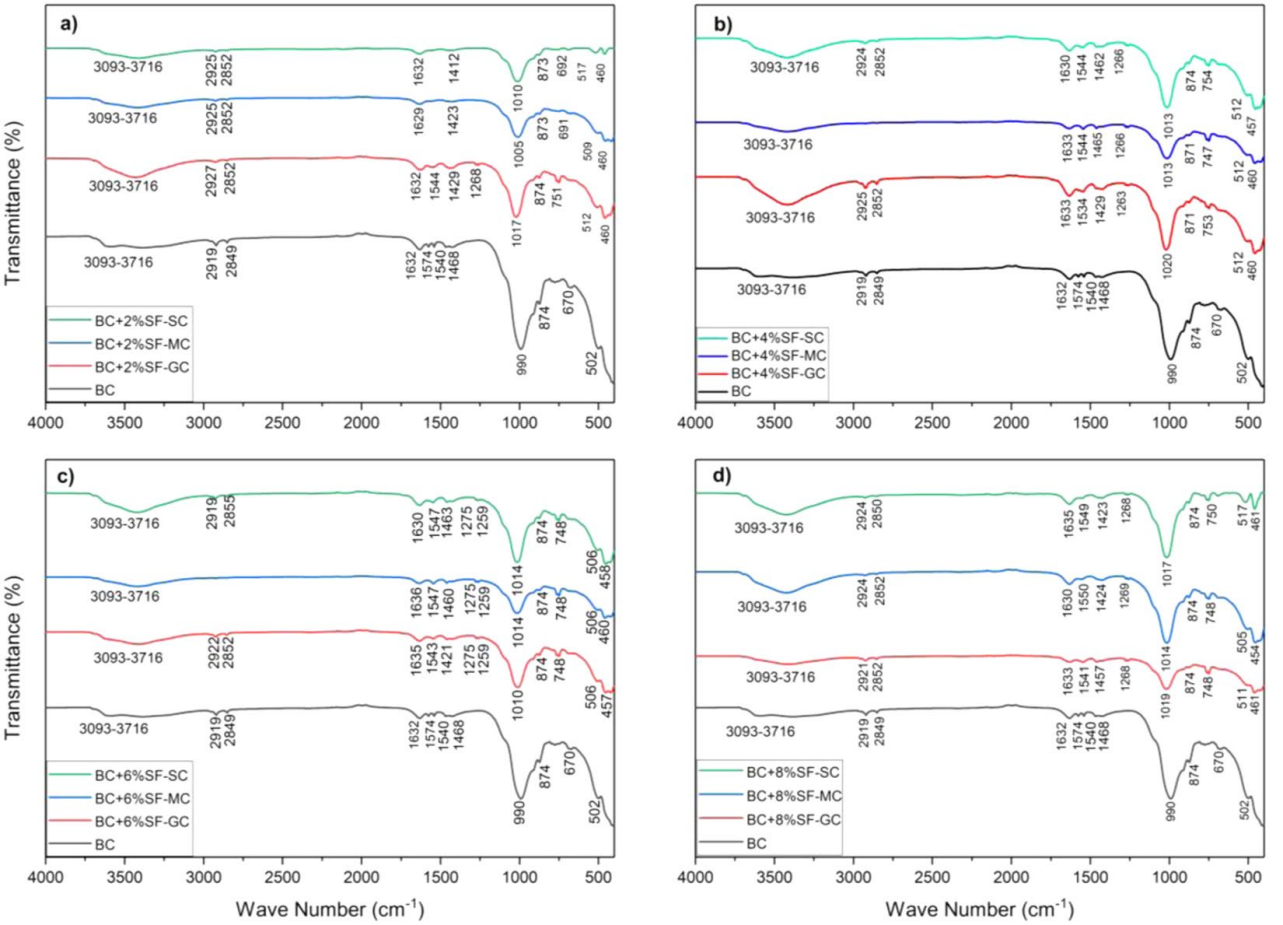
FTIR spectra of the effect of the curing method on treated and untreated expansive soil.

The study of the alternation in the microstructural property of the coupled soil-PP-SF is presented in the study. The expansive soil has been chemically stabilized by SF content with 2%, 4%, 6%, and 8%; hence it alters the chemical bonds of the BC soil. The PP fiber is used as the reinforced material and has no major influence on the chemical structure of BC soil due to inert hydraulic conductive behavior^57^. To assess the chemical alteration formed due to the presence of the SF content, ATR-FTIR analysis has been carried out. The FTIR spectra for the 7, 14, 28 days GC, MC, and SC cured specimens are shown in Fig. 4. The spectra of each concentration of SF and method of curing has been compared with the BC soil. The range of 500 cm^−1^ to 1200 cm^−1^ presented the presence of minerals, 1200 cm^−1^ to 3000 cm^−1^ organic matters, and 3500 cm^−1^ to 4000 cm^−1^ clay minerals^76^.

The characteristic bands of 3093 cm^−1^ - 3716 cm^−1^ represent the vibration (stretching) of the hydroxyl groups (OH) of illite and kaolinite^77^. The peak of the hydroxyl group (OH) of illite and kaolinite has been observed similarly in all the sample ID. A similar trend in the specimens has been observed for peak presented at the 3434 cm^−1^ that attributed to the OH of water. It is observed that with the addition of the SF the 3093 cm^−1^–3716 cm^−1^ band seems to be increased, the maximum stretch for the 8% SF content during submerged curing as shown in Fig. 4(d). The broad band at 3455 cm^−1^ signifies the presence of hydrating material (C-S-H & C-A-S-H)^78^.

The broad IR bond obtained due to the interaction of free dissolved Ca species from SF and forming C-S-H gel. This will also offer a nucleation site for geopolymer gel growth and additional binding material^79^. However, minimal changes have been observed in the gunny bag curing, and it shows the less formation of C-S-H gel, it also conforms with the low calcite content observation in Fig. 3(d). The FTIR spectra show the stretched peak at 874 cm^−1^ and 1468 cm^−1^, ascertaining the presence of carbonate content (C-O bond). With the increase in SF content, the intensity of the band has been decreased, depicting reduction of carbonate group due to the presence of SF content^80^. The splitting in the carbonation band also observed near 1468 cm^−1^ to 1430 cm^−1^ due to partial carbonation of C-S-H gel in the air atmosphere^81^. The carbonation band can be attributed due to the formation of calcium carbonates^82^. The single peak located at 874 cm^−1^ denotes a stretch of OH present in the molecular water and Al-O-H deformation of O-H^83^. The doublet peak at 798 cm^−1^ and 782 cm^−1^ and a single peak located at 694 cm^−1^ can be attributed to the quartz vibrations^84^. The sharp and strong band in the range 990 cm^−1^–1020 cm^−1^ is due to the Si-O stretching vibration. The antisymmetric in-plane Si-O stretching vibration gives a peak at 990 cm^−1^–1012 cm^−1^ and the in-plane stretching band obtained at 1100 cm^−1^ is of Si-O^85^. The peak obtained in the BC soil at 3623 cm^−1^ denoted the presence of montmorillonite domains^85^. The low-intensity peak has been observed in the BC soil, and this peak in missing in all other samples with increasing the curing period. This signifies the reduction of the montmorillonite mineral with the SF inclusion. symmetric stretching vibration (Si–O–Si) at 670 cm^−1^ and 502 cm^−1^ and bending vibration (Si–O–Si and O–Si–O) at about 457 cm^−1–461^ cm^−1^.The other bands at 1008 cm^−1^, and 1104 cm^−1^ are those of Si-O bonds, and the bands at 714 cm^−1^ and 528 cm^−1^ arise from the deformation vibrations of Al_IV_-OSi and Al_VI_-OSi.

### Effect of curing method on the mineralogy of treated and untreated expansive soil

**Figure 5:**
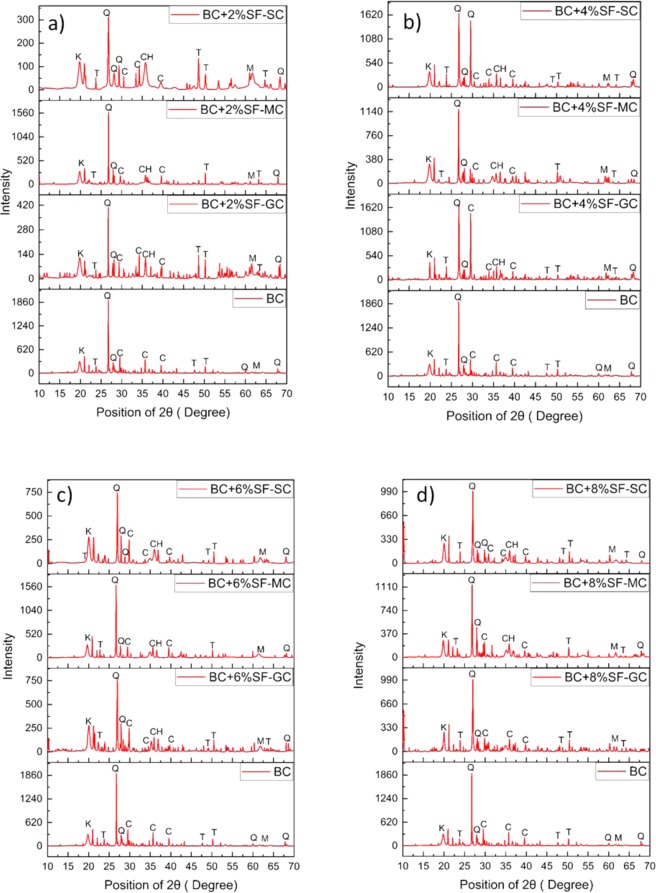
XRD spectra of the effect of the curing method on treated and untreated expansive soil.

The 7, 14, and 28-days crystalline phases for SF stabilized BC soil with GC, MC, and SC curing determined by X-ray diffraction (XRD) analysis, are presented in Fig. 5. The XRD spectra indicate the presence of hydration product i.e., Calcium Silicate Hydrates (C-S-H), Calcium Silicate Aluminates Hydrations (CASH), and composed of portlandite (CH). The analysis of the XRD pattern is shown in Fig. 5 and was obtained using the Pananalytical X′pert Highscore Plus software. X-ray diffraction analysis data shows the peak of quartz as a distinct peak.

XRD analysis depicts the formation of Tobermorite, a mineral formed due to the pozzolanic reaction of the silica fume. The calcium hydrate peak is around 74°, which is the result of the hydration of cement. Calcium hydroxide help in increasing the pH of the soil-SF matrix, which further enhances the pozzolanic reaction. The peak at 60° shows the presence of the quartz; with the increase in the stabilization agent SF and curing period. The decrease in the peak can be caused due to the consumption of the quartz mineral in the pozzolanic reaction. As a result of the formation of the tobermorite mineral formed^86^.The intensity of tobermorite mineral peak the SC cured specimens with 28 days is observed highest. With this, it is ascertained that the formation of hydrated gel during SC curing takes place at higher concentrations. A similar trend has been observed for the MC curing; however, a minimal effect has been seen in GC curing.

### Microstructural coupling effect of PP fiber and SF content on BC soil

**Figure 6:**
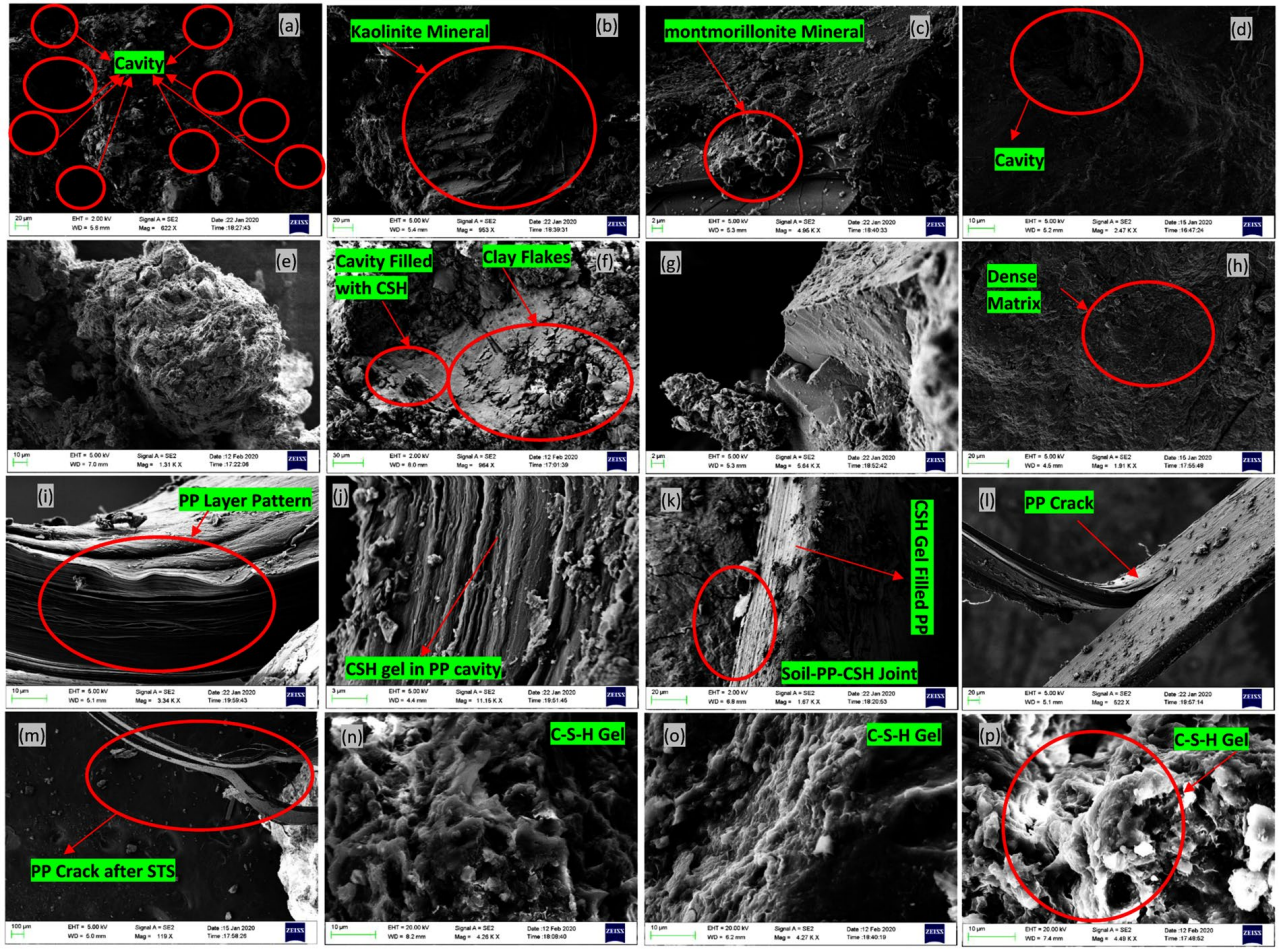
Microstructural Analysis of PP-SF-Soil matrix using SEM.

The coupling effect of PP fiber-reinforced and SF stabilized BC soil on unconfined compressive strength and split tensile strength has its explanation in a microstructural mechanism that can be captured through FE-SEM analysis. Figure 6 presents the microstructural arrangement of the BC Soil-PP fiber and SF. Figure 6(a) shows the microstructure of the BC soil at 20 um, and it can be observed that the various cavities are present in the sample. The SEM images presented in Fig. 6(c) shows irregular wavy edges of the particles and the flaky structure of montmorillonite^87^. Figure 6(b) shows the flat-lying plates typical of kaolinite, whereas Fig. 6(f) depicts expanded, flared, “cornflake” or “oak leaf” texture of Na-montmorillonite^88^. The Fig. 6(h) shows the image formation of the sample ID BC+ 8%SF; it can be observed very dense soil matrix between the soil particle even at the 20 um. This shows the formation of the CSH gel filled the cavity present in the BC soil and formed a dense matrix. This result also supported by the spectra obtained for the FTIR and XRD in Figs. 4 and 5 respectively. Figure 6(e) shows the interesting result of the formation of C-S-H gel on the clay mineral and covering the soil particle. The gel formed on the clay particle also reduces the over consolidation behavior of expansive soil^89^. Figure 6(i) gives the microstructure of the PP fiber, and the various cavities are present in the PP fiber layered structure, and it can cause a failure plane during the compression loading. With the formation of C-S-H gel, the cavity of PP fiber filled with the produced gel and stiffed PP microstructure formed, as shown in Fig. 6(j,k). After STS tests the fiber break, as shown in Fig. 6(m), however, it can be observed that fiber reinforcement helps to bind the clay structure. Figure 6(l) shows the presence of CSH gel makes PP fiber stiffer; as a result, it gives higher tensile strength. The formation of C-S-H gel with the 8% SF content with three different curing methods i.e., GC, MC, and SC curing, has been showing in Fig. 6(n–p). The formation of C-S-H gel is higher for the SC curing method; however, very stiff and homogeneous formation has been observed due to MC curing. MC curing maintains the temperature and humidity at the constant level and formation takes place in homogeneous nature. Figure 6(k) also shows the interaction of PP and Soil, the connection seems very strong and filled with the gel. This behavior can be attributed due to soil- PP fiber interaction with C-S-H gel and as a result, strong bond observed.

### Effect of SF content on the chemical element formation in BC Soil

**Figure 7:**
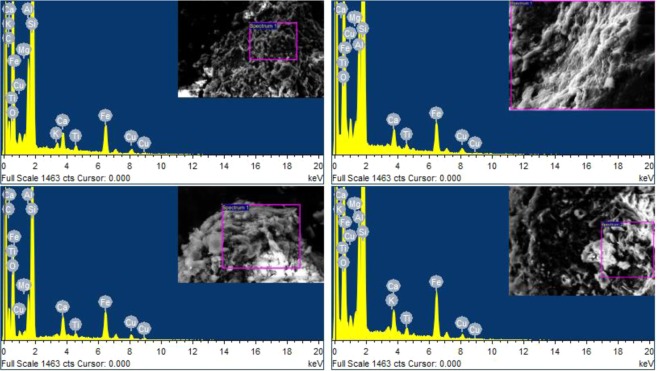
EDX spectra of treated and untreated expansive soil.

Figure 7 shows the EDX analysis of the selected SEM images for 2%, 4%, 6%, and 8% SF content. The intensity of calcium peak is dominant in all sections, which is usually expected due to the development of a hydration reaction. However, in all the cases, the intensity of the silica peak likely represents the existence of a pozzolanic effect^90^. This is mostly followed by the peaks of calcium and alumina. The 8% SF content shows the highest intensity of silica peak that offers more favorable UCS reponse^90^. The elements identified by the EDX test were confirmed with the results obtained for the FTIR and XRD as shown in Figs. 4 and 5. In general, major crystalline phases that occurred in the CSH gel matrix of SF stabilized BC soil specimens are responsible for the strength. The UCS performance of the specimens is well connected with the pattern of spectra obtained in the EDX. In general, for an increasing UCS value of SF stabilized BC soil, higher amounts of Si, and a sufficient amount of Ca and Al become appropriate conditions. The formation of the aluminosilicate gel or calcium silicate hydrate gel is produced due to the pozzolanic reaction of SF stabilized BC soil specimens, which provides dense structure^91^.

### Effect of curing method on UCS and UPV of treated and untreated BC soil under F-T cycle

**Figure 8:**
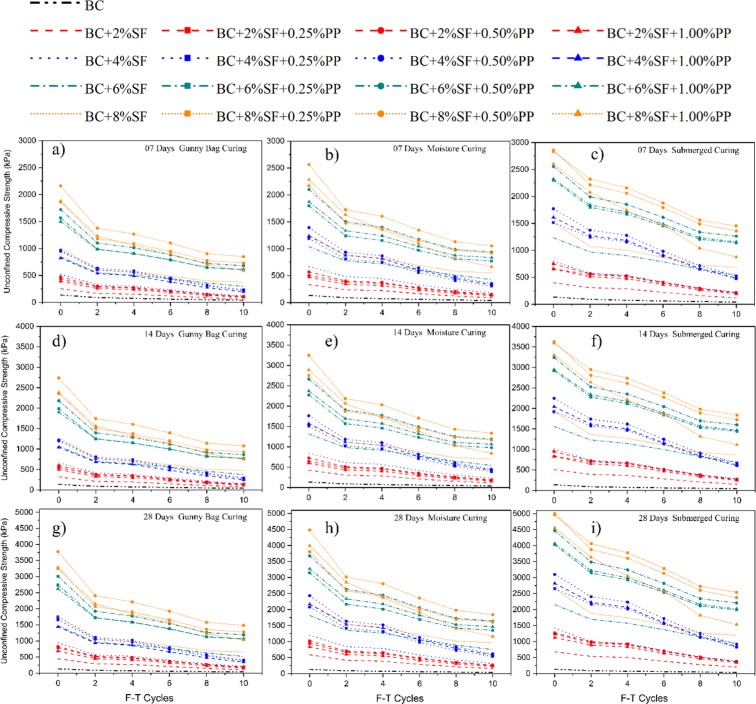
Effect of curing method on UCS of treated and untreated BC soil under F-T cycle.

The effect of the curing method on the unconfined compressive strength of the PP fiber-reinforced and SF stabilized expansive soil is shown in Fig. 8 under the freezing-thawing cycle. It has been observed that with increasing the SF content, the values of UCS increase exponentially with respect to the BC soil UCS value, as shown in Fig. 2. In order to assess the durability of soil- PP fiber and SF matrix F-T cycle tests have been carried out. As observed in Fig. 3(d), the increase of the UCS strength can be attributed to the formation of C-S-H/ C-A-H gel.

The formation of a hydrated gel fills the pore in the soil cavities, and it surrounded soil particles to form a relatively stable spatial network structure^70^. The dense matrix formed due to the formation of hydrated gel increase stiffness and strength of stabilized specimens^67^. The freezing-thawing action induced a swelling-shrinkage behavior in the treated and untreated specimens. During the thawing cycle, the volume of the specimens increased, and while undergoing the freezing, the volume shrinks. Due to this non-recurrence volume change, the density decrease and caused a tangible reduction in the strength^92^. The average loss in the UCS was observed 40%, 33.5% and 30.0% for the GC, MC, and SC, respectively. Figure 8(a) shows the effect of F-T cycles on GC cured specimens, and it is observed that at the lower concentration of SF, i.e., 2% and 4%, the UCS decreased exponentially.

During the SC curing, since the water is infiltrated in the entire specimen and a pozzolanic reaction takes place in the entire specimen homogenously. The homogenous distribution of the C-S-H gel produced a definite soil-PP fiber matrix and gave higher strength. The SC cured specimens retain the maximum strength during F-T cycles. After the 8^th^ cycle of the F-T, almost similar results were observed, and minimal influence noticed on the specimen. The specimen with 8%SF and 0.50%PP shows the highest UCS value at the 10^th^ F-T cycle, although, it lost almost 51.23% strength. At the same time, the specimens with 4%SF and 0.50%PP lost only 27.21% strength at the 10^th^ F-T cycle.

From these results, it can be concluded that with the increase in the higher concentration of SF, a more hydrated gel formed in the specimens. The hydrated gel formed, broke due to swelling -shrinkage behavior and with higher concentration lose more strength^93^. However, no significant influence of PP fiber has been observed with increasing the F-T cycle. It reflects the inert behavior of the PP fiber with temperature variation and can be considered as the more durable material. The 7 days of cured specimens have lost more strength during F-T cycles, which depicts the lower concentration of hydrated gel formation. With the increasing, the curing period, the stabilized specimens show effective in durability.

**Figure 9:**
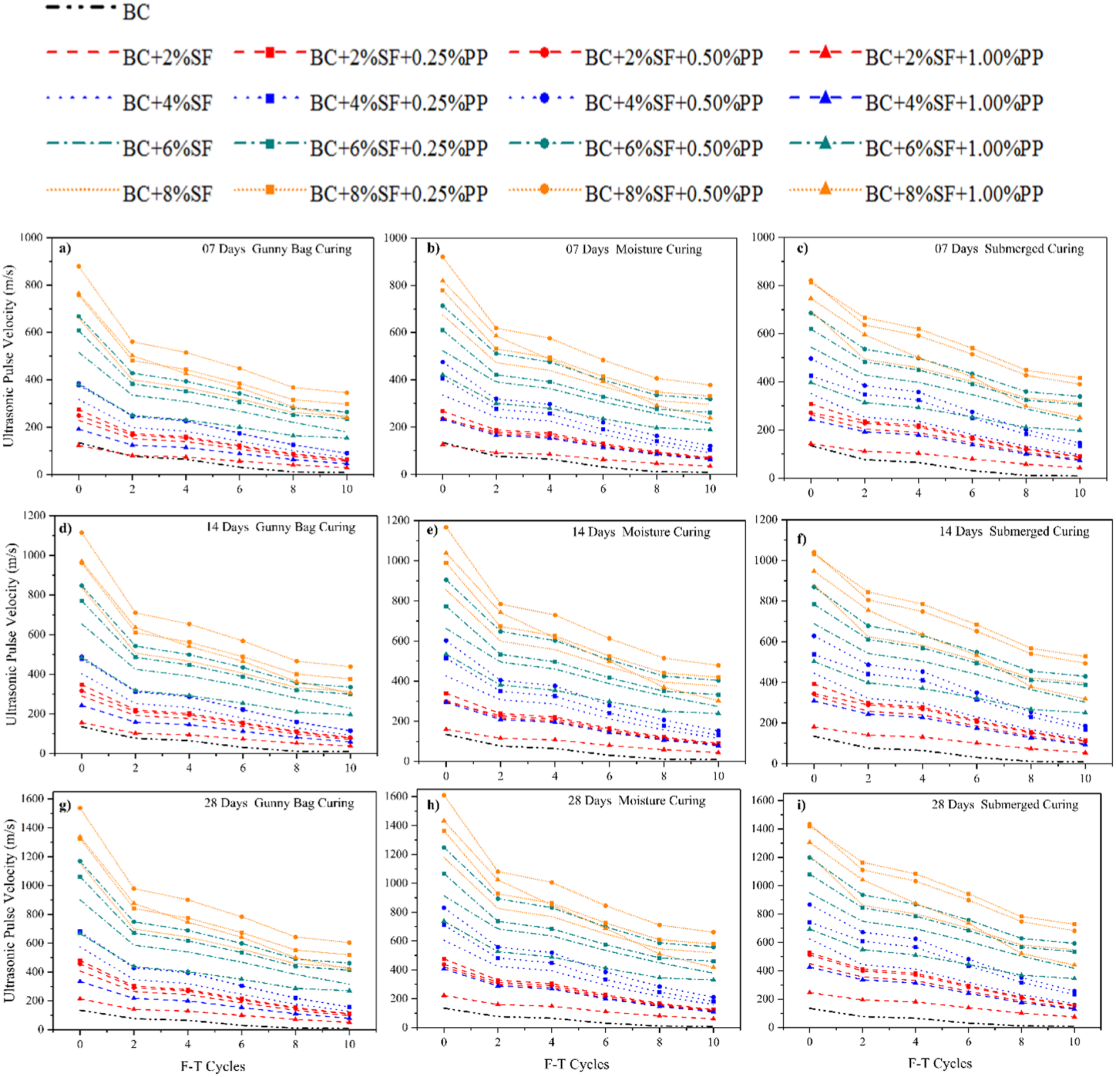
Effect of curing method on UPV of treated and untreated BC soil under F-T cycle.

The effect of the curing method on the ultrasonic pulse velocity test (UPV) of the PP fiber-reinforced and SF stabilized expansive soil is shown in Fig. 9 under the freezing-thawing cycle. The UPV of the specimens has been calculated after every alternate F-T cycle, i.e., 0,2,4,6,8,10 cycles before UCS and STS tests. The UPV value has decreased with the increase in the F-T cycles and process a similar trend, as observed in UCS results, as shown in Fig. 8. The UPV values have decreased due to the breaking of the hydrated CSH/CASH gel during the F-T cycle, and as a result, the treated specimens lose the strength^90^. The UPV value can be used to predict the durability of the SF stabilized BC soil reinforced with PP fiber. From the experimental results, it is concluded with the reduction in the UCS value, the value of UPV also decreased.

### Effect of curing method on STS of treated and untreated BC soil under F-T Cycle

**Figure 10:**
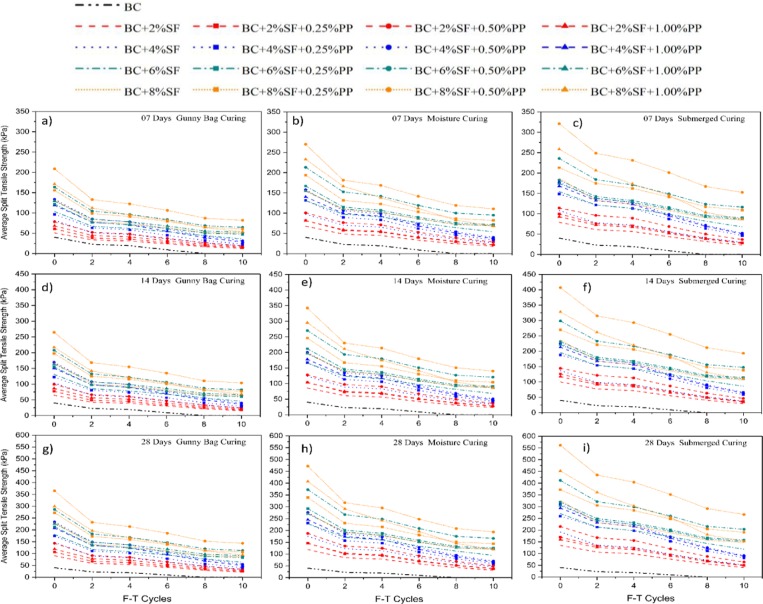
Effect of curing method on STS of treated and untreated BC soil under F-T cycle.

The split tensile strength (STS) test has been carried out under F-T cycles, and the results are shown in Fig. 10. The inclusion of the SF content processes the hydrated gel in the treated specimens, and as a result, brittle shear failure is observed. The brittle shear failure can cause severe damages during the cyclic loading; hence the tensile strength plays a vital role in the performance of the stabilized BC soil. From Fig. 10(a), it is observed that the higher concentration of the PP fiber content i.e., 0.50%, and 1.00% induced higher tensile strength. The submerged curing specimens having higher tensile strength, i.e., 321.17 kPa, 407.07 kPa, and 561.47kPa for 7, 14, 28 days, respectively. During the F-T cycle, it was observed that initially specimen with 8%SF and 1.00%PP are having more STS value than the specimen with 6%SF and 0.5%PP. However, after the 8^th^ cycle of F-T, the STS value of 6%SF and 0.50%PP specimens were increased.

From the results, it can be concluded that the higher concentration of the PP requires more bond strength of the C-S-H gel, and once it broke during the F-T cycle, the value of the tensile strength decreased^94^. The curing method shows the same trend as indicated in the UCS and UPV, as presented in Figs. 8 and 9, respectively. After the 6^th^ F-T cycle, the untreated soil specimen was failed, and the result of STS values has been reported as zero.

## Conclusion

The layered structure of the clay minerals and cations adsorbed for the charged equilibrium induced swelling-shrinkage in expansive soil. A higher nonrecurring swelling-shrinkage behavior of expansive soil culminate damages to the built infrastructure. This study presents the micro-physical investigation of polypropylene (PP) fiber-reinforced, and silica fume (SF) stabilized expansive soil (BC) subgrade. The coupling effect of soil, PP fiber, and SF has been explored under the freezing-thawing (F-T) cycle to assess the durability of treated BC Soil. The effect of different curing methods, i.e., GC, MC, and SC, to a period of 7, 14, and 28 days has been considered. All the experiments have been conducted at the laboratory; therefore, before implementing the proposed proportion of SF and PP fiber, the large scale field testing needs to be carried out. Based on the results and discussion presented, the following conclusions were made:

- The SF possess similar property like cement and can be used as an alternative to BC soil stabilization. With the inclusion of SF content in the BC soil, hydrated gel CSH, and/or CASH formed, which fills the cavity and dense stabilized soil matrix. The filled densified soil structure has been observed in the SEM micrographs.
- XRD analysis depicts the formation of Tobermorite, a mineral formed due to the pozzolanic reaction of the silica fume. The values of pH, calcite content, and EC are also increased, which attributed the chemical alteration in the treated soil.
- The curing method and duration staggeringly influence the strength of SF treated BC soil; therefore, three different curing method, i.e., moisture-controlled curing (MC), gunny bag curing (GC), and water submerged curing (SC) to a period of 7, 14, and 28 days were considered. The SC and MC curing method shows an exponentially increase in the UCS and STS values.
- Based on the F-T test, it can be concluded that the PP fiber having higher durability and can be used for controlling the brittle shear failure in chemically stabilized soil. Due to the high specific surface area of SF, it fills the cavities of the untreated BC soil and produced a dense soil and PP fiber a matrix due to the pozzolanic reaction as ascertained from the results of the microstructure. However, due to non-recurrence volume change during the F-T cycle, the STS and UTS values reduced.
- Based on the mechanical properties of the treated BC soil specimen, the results of the MC and SC curing are higher and can be considered as the effective curing method. The maximum tensile and compressive strength has been observed for the with 8%SF and 0.50%PP; therefore, 8% SF and 0.50% PP combination matrix can be considered as the optimum proportion for the stabilization and furthers for the field application study.

## References

[1] Fouzia Mebarki. *Composite materials based on recycled polyethylene terephthalate and polyethylene naphthalate for electrical applications*, http://espace.etsmtl.ca/1955/1/MEBARKI _Fouzia_thèse.pdf (2017).

[2] Thirumalai R, Babu SS, Naveennayak V, Nirmal R, Lokesh G (2017). A Review on Stabilization of Expansive Soil Using Industrial Solid Wastes. Engineering.

[3] Shi B, Jiang H, Liu Z, Fang HY (2002). Engineering geological characteristics of expansive soils in China. Eng. Geol..

[4] Dasog, G. S. & Mermut, A. R. Expansive soils and clays. in *Encyclopedia of Earth Sciences Series* 297–300, 10.1007/978-1-4020-4399-4_124 (Springer Netherlands, 2013)..

[5] Thomas, P. J. Quantifying Properties and Variability of Expansive Soils in Selected Map Units. (Virginia Polytechnic Institute and State University, 1998).

[6] Khazaei J, Moayedi H (2019). Soft Expansive Soil Improvement by Eco-Friendly Waste and Quick Lime. Arab J Sci Eng.

[7] Puppala, A. J., Pedarla, A., Pino, A. & Hoyos, L. R. Diffused double-layer swell prediction model to better characterize natural expansive clays. *J. Eng. Mech*. **143** (2017).

[8] Steinberg, M. L. *Geomembranes and the control of expansive soils in construction*. (McGraw-Hill, 1998).

[9] Puppala AJ, Wattanasanticharoen E, Punthutaecha K (2003). Experimental evaluations of stabilisation methods for sulphate-rich expansive soils. Gr. Improv..

[10] Kumar P, Singh SP (2008). Fiber-Reinforced Fly Ash Subbases in Rural Roads. J. Transp. Eng..

[11] Moayedi, H. & Nazir, R. Malaysian Experiences of Peat Stabilization, State of the Art. *Geotechnical and Geological Engineering* vol. 36 (2018).

[12] Zhang M (2015). Calcium-free geopolymer as a stabilizer for sulfate-rich soils. Appl. Clay Sci..

[13] Tataranni, P. *et al*. A laboratory and field study on 100% Recycled Cement Bound Mixture for base layers. *Int*. *J. Pavement Res. Technol*., 10.1016/j.ijprt.2017.11.005 (2018).

[14] Rojas E, Romo MP, Garnica P, Cervantes R (2006). Analysis of deep moisture barriers in expansive soils. II: Water flow formulation and implementation. Int. J. Geomech..

[15] Rojas E, Romo MP, Cervantes R (2009). Closure to “Analysis of Deep Moisture Barriers in Expansive Soils. I: Constitutive Model Formulation” by Eduardo Rojas. Miguel P. Romo, and Refugio Cervantes. Int. J. Geomech..

[16] Soundara B, Robinson RG (2009). Influence of test method on swelling pressure of compacted clay. Int. J. Geotech. Eng..

[17] Li, M., Fang, C., Kawasaki, S. & Achal, V. Fly ash incorporated with biocement to improve strength of expansive soil., 10.1038/s41598-018-20921-0. 10.1038/s41598-018-20921-0 PMC5803229 29416093

[18] Liu Y (2019). Utilization of Cementitious Material from Residual Rice Husk Ash and Lime in Stabilization of Expansive Soil. Adv. Civ. Eng..

[19] Ikeagwuani CC, Nwonu DC (2019). Emerging trends in expansive soil stabilisation: A review. J. Rock Mech. Geotech. Eng..

[20] Sharma, M., Satyam, N. & Reddy, K. R. Investigation of various gram-positive bacteria for MICP in Narmada Sand, India. *Int*. *J. Geotech. Eng*. 1–15, 10.1080/19386362.2019.1691322 (2019).

[21] Selvakumar S, Soundara B (2019). Swelling behaviour of expansive soils with recycled geofoam granules column inclusion. Geotext. Geomembranes.

[22] Shukla, S. K. *An Introduction to Geosynthetic Engineering*. *An Introduction to Geosynthetic Engineering*, 10.1201/9781315378930 (CRC Press, New York, 2016).

[23] Tiwari, N. & Satyam, N. An experimental study on the behavior of lime and silica fume treated coir geotextile reinforced expansive soil subgrade. *Eng. Sci. Technol. an Int. J*., 10.1016/j.jestch.2019.12.006 (2020).

[24] Phanikumar BR, Nagaraju TV (2018). Effect of Fly Ash and Rice Husk Ash on Index and Engineering Properties of Expansive Clays. Geotech. Geol. Eng..

[25] Mirzababaei M, Yasrobi S, Al-Rawas A (2009). Effect of polymers on swelling potential of expansive soils. Proc. Inst. Civ. Eng. - Gr. Improv..

[26] Tiwari N, Satyam N (2019). Experimental Study on the Influence of Polypropylene Fiber on the Swelling Pressure Expansion Attributes of Silica Fume Stabilized Clayey Soil. Geosciences.

[27] Jha AK, Sivapullaiah PV (2017). Physical and strength development in lime treated gypseous soil with fly ash — Micro-analyses. Appl. Clay Sci..

[28] Elert K, Azañón JM, Nieto F (2018). Smectite formation upon lime stabilization of expansive marls. Appl. Clay Sci..

[29] Punthutaecha K, Puppala AJ (2014). P. E. S. K. V. and H. I. Volume Change Behaviors of Expansive Soils Stabilized with Recycled Ashes and Fibers..

[30] Rajkumar, M. R. Recent Advances in Materials, Mechanics and Management. in *3rd International Conference on**Materials, Mechanics and Management* 450 (2017).

[31] Jin L (2014). Combined overexpression of genes involved in pentose phosphate pathway enables enhanced d-xylose utilization by Clostridium acetobutylicum. J. Biotechnol..

[32] Jha AK, Sivapullaiah PV (2016). Volume change behavior of lime treated gypseous soil - influence of mineralogy and microstructure. Appl. Clay Sci..

[33] Taha R (2004). An overview of waste materials recycling in the Sultanate of Oman. Resour. Conserv. Recycl..

[34] Blackburn, R. S. (Richard S.. *Biodegradable and sustainable fibres*. (Woodhead Pub. in association with the Textile Institute, 2005).

[35] Mazzoni, G., Virgili, A. & Canestrari, F. Influence of different fillers and SBS modified bituminous blends on fatigue, self-healing and thixotropic performance of mastics. *Road Materials and Pavement Design*, 10.1080/14680629.2017.1417150 (2017).

[36] Wilberforce T, Baroutaji A, Soudan B, Al-Alami AH, Olabi AG (2019). Outlook of carbon capture technology and challenges. Sci. Total Environ..

[37] Mastali, M. & Abdollahnejad, Z. Carbon dioxide sequestration on fly ash/waste glassalkali-based mortars with recycled aggregates: Compressive strength, hydration products, carbon footprint, and cost analysis. *Carbon Dioxide Sequestration Cem. Constr. Mater*. 299–348, 10.1016/B978-0-08-102444-7.00013-7 (2018)..

[38] Schneider M, Romer M, Tschudin M, Bolio H (2011). Sustainable cement production-present and future. Cement and Concrete Research.

[39] Zhang Z, Zhang B, Yan P (2016). Comparative study of effect of raw and densified silica fume in the paste, mortar and concrete. Constr. Build. Mater..

[40] Asavapisit S, Nanthamontry W, Polprasert C (2001). Influence of condensed silica fume on the properties of cement-based solidified wastes. Cem. Concr. Res..

[41] Koksal F, Gencel O, Kaya M (2015). Combined effect of silica fume and expanded vermiculite on properties of lightweight mortars at ambient and elevated temperatures. Constr. Build. Mater..

[42] Benaicha M, Roguiez X, Jalbaud O, Burtschell Y, Alaoui AH (2015). Influence of silica fume and viscosity modifying agent on the mechanical and rheological behavior of self compacting concrete. Constr. Build. Mater..

[43] Cai G, Liu S, Du Y, Zhang D, Zheng X (2015). Strength and deformation characteristics of carbonated reactive magnesia treated silt soil. J. Cent. South Univ..

[44] Muller ACA, Scrivener KL, Skibsted J, Gajewicz AM, McDonald PJ (2015). Influence of silica fume on the microstructure of cement pastes: New insights from 1H NMR relaxometry. Cem. Concr. Res..

[45] Tang CS, Shi B, Zhao LZ (2010). Interfacial shear strength of fiber reinforced soil. Geotext. Geomembranes.

[46] Yang G, Duan J, Qiu M, Zhou H (2018). Mechanical properties of new waterproof materials and its application in railway subgrade. Zhongnan Daxue Xuebao (Ziran Kexue Ban)/Journal Cent. South Univ. (Science Technol..

[47] Mirzababaei M, Arulrajah A, Horpibulsuk S, Soltani A, Khayat N (2018). Stabilization of soft clay using short fibers and poly vinyl alcohol. Geotext. Geomembranes.

[48] Senol, A., Khosrowshahi, S. K.. & Yildirim, H.. Improvement of Expansive Soils Using Fiber Materials. in The 11th international congress on advances in Civil Engineering (ACE 2014) (2014).

[49] Phanikumar BR, Singla R (2016). Swell-consolidation characteristics of fibre-reinforced expansive soils. Soils Found..

[50] Ma, Q. Y., Cao, Z. M. & Yuan, P. Experimental Research on Microstructure and Physical-Mechanical Properties of Expansive Soil Stabilized with Fly Ash, Sand, and Basalt Fiber. *Adv. Mater. Sci. Eng*. **2018** (2018).

[51] Gao L (2015). Experimental Study on Unconfined Compressive Strength of Basalt Fiber Reinforced Clay Soil. Adv. Mater. Sci. Eng..

[52] Shahinur, S. & Hasan, M. Jute/Coir/Banana Fiber Reinforced Bio-Composites: Critical Review of Design, Fabrication, Properties and Applications. in *Reference Module in Materials Science and Materials Engineering*, 10.1016/B978-0-12-803581-8.10987-7 (Elsevier, 2019).

[53] Orue A (2016). The effect of alkaline and silane treatments on mechanical properties and breakage of sisal fibers and poly(lactic acid)/sisal fiber composites. Compos. Part A Appl. Sci. Manuf..

[54] Putman, B. J. & Amirkhanian, S. N. Utilization of waste fibers in stone matrix asphalt mixtures. In *Resources, Conservation and Recycling* vol. 42 265–274 (2004).

[55] Zou, W. L., Wang, X. Q. & Vanapalli, S. K. Experimental evaluation of engineering properties of GFRP screw anchors for anchoring applications. *J. Mater. Civ. Eng*. **28**, (2016).

[56] Bekhiti M, Trouzine H, Rabehi M (2019). Influence of waste tire rubber fibers on swelling behavior, unconfined compressive strength and ductility of cement stabilized bentonite clay soil. Constr. Build. Mater..

[57] Liu HF (2019). Experimental study on three-dimensional swelling pressure of highly expansive clay in Handan district of China. *Yantu Gongcheng Xuebao/Chinese J*. Geotech. Eng..

[58] EsmaeilpourShirvani N, TaghaviGhalesari A, Khaleghnejad Tabari M, Janalizadeh Choobbasti A (2019). Improvement of the engineering behavior of sand-clay mixtures using kenaf fiber reinforcement. Transp. Geotech..

[59] Ilyas, R. A. *et al*. Sugar palm (Arenga pinnata [Wurmb.] Merr) starch films containing sugar palm nanofibrillated cellulose as reinforcement: Water barrier properties. *Polym. Compos*., 10.1002/pc.25379 (2019).

[60] Chaduvula U, Viswanadham BVS, Kodikara J (2017). A study on desiccation cracking behavior of polyester fiber-reinforced expansive clay. Appl. Clay Sci..

[61] Malekzadeh M, Bilsel H (2012). Swell and Compressibility of Fiber Reinforced Expansive Soils. *Int*. J. Adv. Technol. Civ. Eng..

[62] Kalkan E, Akbulut S (2004). The positive effects of silica fume on the permeability, swelling pressure and compressive strength of natural clay liners. Eng. Geol..

[63] Tiwari, N. & Satyam, N. Experimental study on the influence of polypropylene fiber on the swelling pressure expansion attributes of silica fume stabilized clayey soil. *Geosci*. **9**, (2019).

[64] Wang, Y. *et al*. Behavior of fiber-reinforced and lime-stabilized clayey soil in triaxial tests. *Appl. Sci*. **9** (2019).

[65] Elsharief AM, Zumrawi MME, Salam AM (2014). Experimental Study of Some Factors Affecting Swelling Pressure. Univ. Khartoum Eng. J..

[66] Muller ACA, Scrivener KL, Skibsted J, Gajewicz AM, McDonald PJ (2015). Influence of silica fume on the microstructure of cement pastes: New insights from 1H NMR relaxometry. Cem. Concr. Res..

[67] Shi B, Wu Z, Inyang H, Chen J, Wang B (1999). Preparation of soil specimens for SEM analysis using freeze-cut-drying. Bull. Eng. Geol. Environ..

[68] Goldstein, J. I. *et al*. Coating and Conductivity Techniques for SEM and Microanalysis. in *Scanning Electron Microscopy and X-Ray Microanalysis* 671–740, 10.1007/978-1-4613-0491-3_13 (Springer US, 1992).

[69] Lu Y (2016). Fractal analysis of cracking in a clayey soil under freeze-thaw cycles. Eng. Geol..

[70] Lu, Y. *et al*. Volume changes and mechanical degradation of a compacted expansive soil under freeze-thaw cycles., 10.1016/j.coldregions.2018.10.008 (2018).

[71] Kuo WT, Shu CY (2015). Effect of particle size and curing temperature on expansion reaction in electric arc furnace oxidizing slag aggregate concrete. Constr. Build. Mater..

[72] Bouziadi F, Boulekbache B, Hamrat M (2016). The effects of fibres on the shrinkage of high-strength concrete under various curing temperatures. Constr. Build. Mater..

[73] Goodarzi AR, Akbari HR, Salimi M (2016). Enhanced stabilization of highly expansive clays by mixing cement and silica fume. Appl. Clay Sci..

[74] Kalkan E (2011). Impact of wetting-drying cycles on swelling behavior of clayey soils modified by silica fume. Appl. Clay Sci..

[75] Ouhadi VR, Yong RN, Goodarzi AR, Safari-Zanjani M (2010). Effect of temperature on the re-structuring of the microstructure and geo-environmental behaviour of smectite. Appl. Clay Sci..

[76] Robertson, A. H. J., Hill, H. R. & Main, A. M. Analysis of Soil in the Field using portable FTIR. in *Soil**Spectroscopy: the present and future of Soil Monitoring* 1–20 (2013).

[77] Farmer, V. C. The Infrared Spectra of Minerals. (Mineralogical Society of Great Britain and Ireland, 1974), 10.1180/mono-4.

[78] Sharma LK, Sirdesai NN, Sharma KM, Singh TN (2018). Experimental study to examine the independent roles of lime and cement on the stabilization of a mountain soil: A comparative study. Appl. Clay Sci..

[79] Temuujin J, van Riessen A, Williams R (2009). Influence of calcium compounds on the mechanical properties of fly ash geopolymer pastes. J. Hazard. Mater..

[80] García Lodeiro I, Fernández-Jimenez A, Palomo A, Macphee DE (2010). Effect on fresh C-S-H gels of the simultaneous addition of alkali and aluminium. Cem. Concr. Res..

[81] Kalinkin, A. M., Kalinkina, E. V., Politov, A. A., Makarov, V. N. & Boldyrev, V. V. Mechanochemical interaction of Ca silicate and aluminosilicate minerals with carbon dioxide. in *Journal of Materials Science* vol. 39, 5393–5398 (2004).

[82] García Lodeiro I, Macphee DE, Palomo A, Fernández-Jiménez A (2009). Effect of alkalis on fresh C-S-H gels. FTIR analysis. Cem. Concr. Res..

[83] Vinod JS, Indraratna B, Al Mahamud MA (2010). Stabilisation of an erodible soil using a chemical admixture. Proc. Inst. Civ. Eng. Gr. Improv..

[84] Christelle, B. Contribution À L’Étude De L’Activation Thermique Du Kaolin: Évolution De La Structure Cristallographique Et Activité Pouzzolanique. 96-98 pp (2005).

[85] Madejová, J., Gates, W. P. & Petit, S. IR Spectra of Clay Minerals. in *Developments in Clay Science* vol. 8, 107–149 (Elsevier B.V., 2017).

[86] Sharma AK, Sivapullaiah PV (2016). Strength development in fly ash and slag mixtures with lime. Proc. Inst. Civ. Eng. Gr. Improv..

[87] Atahu, M. K., Saathoff, F. & Gebissa, A. Strength and compressibility behaviors of expansive soil treated with coffee husk ash. *J. Rock Mech. Geotech. Eng*., 10.1016/j.jrmge.2018.11.004 (2019).

[88] Inoue A, Sciences E, Hampshire N (1986). morphology of Clay Minerals in the Smectite-To-Illite conversion Series By scaning electronic microscopy. Clays Clay Miner..

[89] Al-Taie A, Disfani MM, Evans R, Arulrajah A, Horpibulsuk S (2016). Swell-shrink Cycles of Lime Stabilized Expansive Subgrade. Procedia Eng..

[90] Canakci, H., Güllü, H. & Alhashemy, A. Performances of using geopolymers made with various stabilizers for deep mixing. *Materials (Basel)*. **12** (2019). 10.3390/ma12162542 PMC6721069 31405008

[91] Pourabbas Bilondi M, Toufigh MM, Toufigh V (2018). Experimental investigation of using a recycled glass powder-based geopolymer to improve the mechanical behavior of clay soils. Constr. Build. Mater..

[92] Lu, Y., Liu, S., Zhang, Y., Li, Z. & Xu, L. Freeze-thaw performance of a cement-treated expansive soil. *Cold Reg. Sci. Technol*. **170** (2020).

[93] Yang LS (2018). Cracking in an expansive soil under freeze–thaw cycles. Sci. Cold Arid Reg..

[94] Du C, Yang G, Zhang T, Yang Q (2019). Multiscale study of the influence of promoters on low-plasticity clay stabilized with cement-based composites. Constr. Build. Mater..

[95] Bureau of Indian Standard. *IS 2720**(Part I-XL) Methods of Test for Soils*.

[96] ASTM. D747-10: Standard Test Method for Apparent Bending Modulus of Plastics by Means of a Cantilever Beam 1., 10.1520/D0747-10.

[97] Committee, D. ASTM D792-08 Standard Test Methods for Density and Specific Gravity (Relative Density) of Plastics by Displacement., 10.1520/D0792-13 (1900).

[98] ASTM., A. S. for T. and M. Designation (2014). D638-14 Standard test Method for Tensile Properties of Plastics. Astm.

[99] ASTM D7138 - 16 Standard Test Method to Determine Melting Temperature of Synthetic Fibers, https://www.astm.org/Standards/D7138.htm.

[100] ASTM E3020 - 16a Standard Practice for Ignition Sources, https://www.astm.org/Standards/E3020.htm.

[101] ASTM D3800-99. Standard Test Method for Density of High-Modulus Fibers. *ASTM lnternational* **6**, 10.1520/D3800-16 (2016).

[102] ASTM D5103 - 07(2018) Standard Test Method for Length and Length Distribution of Manufactured Staple Fibers (Single-Fiber Test), https://www.astm.org/Standards/D5103.htm.

[103] ASTM C1012 / C1012M - 18b Standard Test Method for Length Change of Hydraulic-Cement Mortars Exposed to a Sulfate Solution, https://www.astm.org/Standards/C1012.htm.

[104] ASTM C563 - 07 Standard Test Method for Approximation of Optimum SO3 in Hydraulic Cement Using Compressive Strength, https://www.astm.org/DATABASE.CART/HISTORICAL/C563-07.htm.

[105] ASTM C1240 - 20 Standard Specification for Silica Fume Used in Cementitious Mixtures, https://www.astm.org/Standards/C1240.

[106] ASTM E1621 - 13 Standard Guide for Elemental Analysis by Wavelength Dispersive X-Ray Fluorescence Spectrometry, https://www.astm.org/Standards/E1621.htm.

